# RNA Sequencing for Gene Expression Profiles in a Rat Model of Middle Cerebral Artery Occlusion

**DOI:** 10.1155/2018/2465481

**Published:** 2018-11-06

**Authors:** Xianchun Duan, Jianghua Gan, Fan Xu, Lili Li, Lan Han, Can Peng, Qiuyu Bao, Ling Xiao, Daiyin Peng

**Affiliations:** ^1^The First Affiliated Hospital of Anhui University of Chinese Medicine, No 117 Meishan Road, Shushan District, Hefei 230031, China; ^2^Anhui University of Chinese Medicine, No 1 Qianjiang Road, Xinzhan District, Hefei 230012, China; ^3^Key Laboratory of Chinese Medicinal Formula Research, Anhui University of Chinese Medicine, Xinzhan District, Hefei 230012, China; ^4^China Pharmaceutical University, No 639 Longmian Avenue, Jiangning District, Nanjing 211198, China

## Abstract

Gene expression regulatory mechanisms in models of middle cerebral artery occlusion (MCAO) have been assessed in some studies, but questions remain. The discovery of differentially expressed genes (DEGs) between MCAO and control rats analyzed by next-generation RNA sequencing is of particular interest. These DEGs may help guide the clinical diagnosis of stroke and facilitate selection of the optimal treatment strategy. Twenty rats were equally divided into control and MCAO groups. Three rats from each group were randomly selected for RNA sequencing analysis. Sequence reads were obtained from an Illumina HiSeq2500 platform and mapped onto the rat reference genome RN6 using Hisat2. We identified 1,007 significantly DEGs with p<0.05, including 785 upregulated (fold change [FC]>2) and 222 downregulated (FC<0.5) DEGs, in brain tissue from MCAO rats compared with that from control rats, and numerous immune response genes were identified. Gene ontology (GO) analysis revealed that the majority of the most enriched and meaningful biological process terms were mainly involved in immune response, inflammatory response, cell activation, leukocyte migration, cell adhesion, response to external stimulus, cell migration, and response to wounding. Also enriched were immune-related pathways and neural-related pathways. Similar to GO molecular function terms, the enriched terms were mainly related to cytokine receptor activity. Six DEGs were verified by quantitative real-time polymerase chain reaction (qRT-PCR). Protein-protein interaction analysis of these hits revealed that MCAO affects complement and coagulation cascades and chemokine signaling pathway. Our study identified novel biomarkers that could help further elucidate MCAO mechanisms and processes.

## 1. Introduction

Ischemic stroke is a leading cause of global mortality and morbidity. Immediately following the event, neurovascular reperfusion to the infarcted area can injure the tissue, resulting in more serious disability [1, 2]. Middle cerebral artery occlusion (MCAO) is a widely used model for studying neuroprotective therapies. For example, it was used to test the effectiveness of thrombolytic therapy [3, 4]. Thrombolysis is one of the most effective and economical treatments for ischemic stroke [5]. It is well known that brain damage can be exacerbated by reperfusion after thrombolysis [6]. Presumably, this secondary injury impacts multiple gene functions. Most existing studies have analyzed the functions of specific genes and signaling pathways. [7–9]. We propose that a broad analysis of gene expression changes could be used to develop better neuroprotectants for cerebral ischemia.

In recent decades, mRNA expression profiling performed by microarray or high-throughput RNA sequencing (RNA-seq) has been used to uncover molecular mechanisms and explore diagnostic and predictive biomarkers, particularly in complicated diseases such as diabetes, cancer, and stroke [10–12]. RNA-seq was applied for global gene expression profiling of the hippocampus following reperfusion with orexin-A [13]. Gaowa et al. performed RNA-seq to investigate transcriptome expression in a rat stroke model treated with the traditional Mongolian medicine Eerdun Wurile [14].

The main aim of this study was to identify protein-coding genes regulation networks 4 days after MCAO. RNA-seq was used to investigate the mRNA profiles in the brain tissue of rats subjected to MCAO. By a series of bioinformatics analyses, many immune-related pathways (B cell receptor signaling pathway, primary immunodeficiency, Fc epsilon RI signaling pathway, natural killer cell-mediated cytotoxicity, chemokine signaling pathway, cytokine-cytokine receptor interaction, complement and coagulation cascades, etc.) and neural-related pathways (neuroactive ligand-receptor interaction, neurotrophin signaling pathway) were identified, and six key genes were verified by quantitative real-time polymerase chain reaction (qRT-PCR).

## 2. Material and Methods

### 2.1. Animal Experiments and Sample Collection

Male Sprague Dawley rats (180-230 g, SPF grade) were obtained from the Experimental Animal Center of Anhui Medical University by Animal Experiments of Anhui university of Chinese medicine. All rats were anesthetized with sodium pentobarbital during all surgical procedures to minimize suffering, and the MCAO and sham surgeries were performed as previously described [15]. Four days after MCAO, the left hemispheres were collected and immediately frozen in liquid nitrogen.

### 2.2. RNA Extraction and Sequencing

Total RNA was extracted using mirVana™ miRNA Isolation kit (Cat# AM1561, Ambion, Foster City, CA) following the manufacturer's instructions. RNA sample quantity and quality were determined using a NanoDrop 2000 (Thermo Fisher Scientific, Waltham, MA) and an Agilent 2100 bioanalyzer (Agilent Technologies, Santa Clara, CA). TruSeq® Stranded Total RNA Sample Preparation kits (Illumina, San Diego, CA) were used to prepare libraries following the manufacturer's instructions. Purified libraries were quantified by a Qubit® 2.0 Fluorometer (Life Technologies, Carlsbad, CA) and Agilent 2100 bioanalyzer. Clusters were generated by cBot with the library and sequenced on an Illumina HiSeq 2500 platform (San Diego, CA). All sequencing was performed at Origin-Biotech Inc. (Ao-Ji Biotech, Shanghai, China).

### 2.3. DEG Analysis

FastQC was conducted for Quality control (QC) of RNA-seq reads (v. 0.11.3) (http://www.bioinformatics.babraham.ac.uk/projects/fastqc). Trimming was performed by seqtk for known Illumina TruSeq adapter sequences, poor reads, and ribosome RNA reads (https://github.com/lh3/seqtk). The trimmed reads were then mapped to the* Rattus norvegicus* reference genome (rno6) by the Hisat2 (version: 2.0.4) [16, 17]. StringTie (version: 1.3.0) was performed for each gene count from trimmed reads [17, 18]. Gene counts were normalized by trimmed mean of M-values (TMM) [19], and fragments per kilobase of transcript per million mapped reads (FPKM) in Perl script [20]. edgeR was performed for determining differential expression genes [21] and threshold with p<0.05 and absolute values of log2 (fold change) >1 [22].

### 2.4. Functional Enrichment Analysis

GO and KEGG pathways were enriched by R package (v 3.5.1) to better understand the functions of the DEGs [23]. In our study, clusterProfiler was applied to analysis of GO terms and KEGG pathways, and the top 30 GOs and pathways are presented [24].

### 2.5. PPI Network Construction and Module Analysis

STRING is a database that provides comprehensive information about interactions between proteins, including prediction and experimental interaction data [25]. In our study, the STRING tool was used to map PPIs among the DEGs considering interactions of combined scores ≥0.4. Then, Cytoscape was used to visualize the network [26]. The PPI network was used to filter modules based on the Molecular Complex Detection plug-in (MCOD) in Cytoscape [27] with standard set following degree cut-off=2, k-core=2, node score cut-off=0.2, and max depth=100. An MCODE score ≥4 and node ≥10 were considered for functional enrichment analysis of the modules. GO and KEGG enrichment for DEGs in the four modules were performed in clusterProfiler.

### 2.6. Validation of Differentially Expressed mRNAs from the qRT-PCR Sequencing Profile

qRT-PCR is the gold standard of mRNA detection and was performed to verify the RNA-seq results. Glyceraldehyde 3-phosphate dehydrogenase (GAPDH) served as the internal control. Relative mRNA expression was determined using the 2-ΔΔCT method. Six genes were analyzed: Top2a, Cdk1, Ccna2, Ccnb1, Th, and Esr1. Brain tissue was sequenced from the MCAO and control groups (n=3/group), and each experiment was performed in triplicate.

## 3. Results

### 3.1. DEG Screening

**Figure 1:**
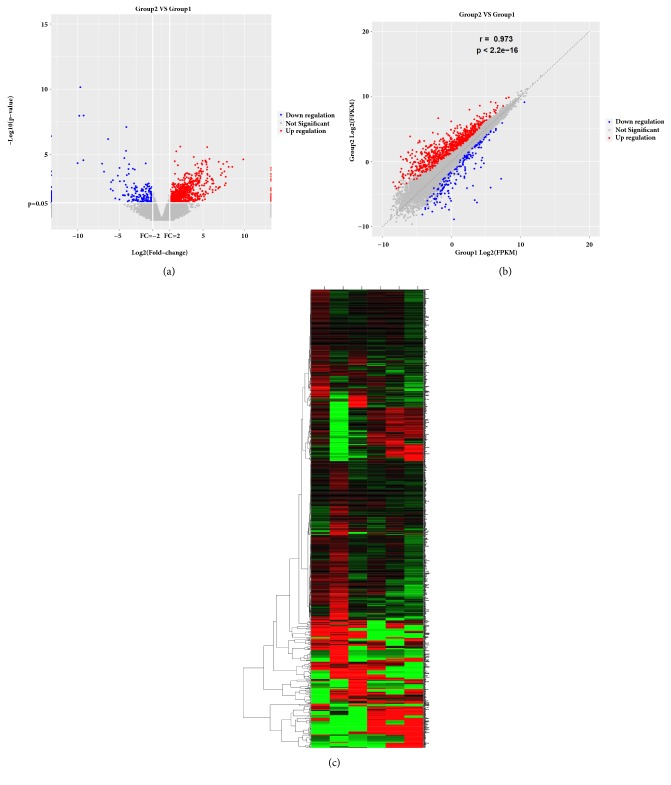
Differentially expressed genes between the MCAO and control groups. (a) DEGs displayed on a volcano plot. Blue and red indicate >twofold decreased and increased expression in MCAO, respectively (p<0.05). Gray indicates no significant difference. (b) Differentially expressed genes were displayed on a scatter plot. Blue and red indicate >twofold decreased and increased expression in MCAO, respectively (p<0.05). Gray indicates no significant difference. (c) Hierarchical clustering; numbers were the samples used for RNA sequencing.

EdgeR software was used to screen DEGs with p<0.05 and |log_2_FC|>1. We identified 1,007 DEGs: 785 upregulated and 222 downregulated. Differential mRNA expression between the MCAO and control groups was represented in volcano and scatter plots (Figures 1(a) and 1(b)). Hierarchical clustering of DEGs was visualized (Figure 1(c)). The top 20 up- and downregulated DEGs are listed in Tables 1 and 2.

### 3.2. GO Functional Enrichment Analysis of DEGs

**Figure 2:**
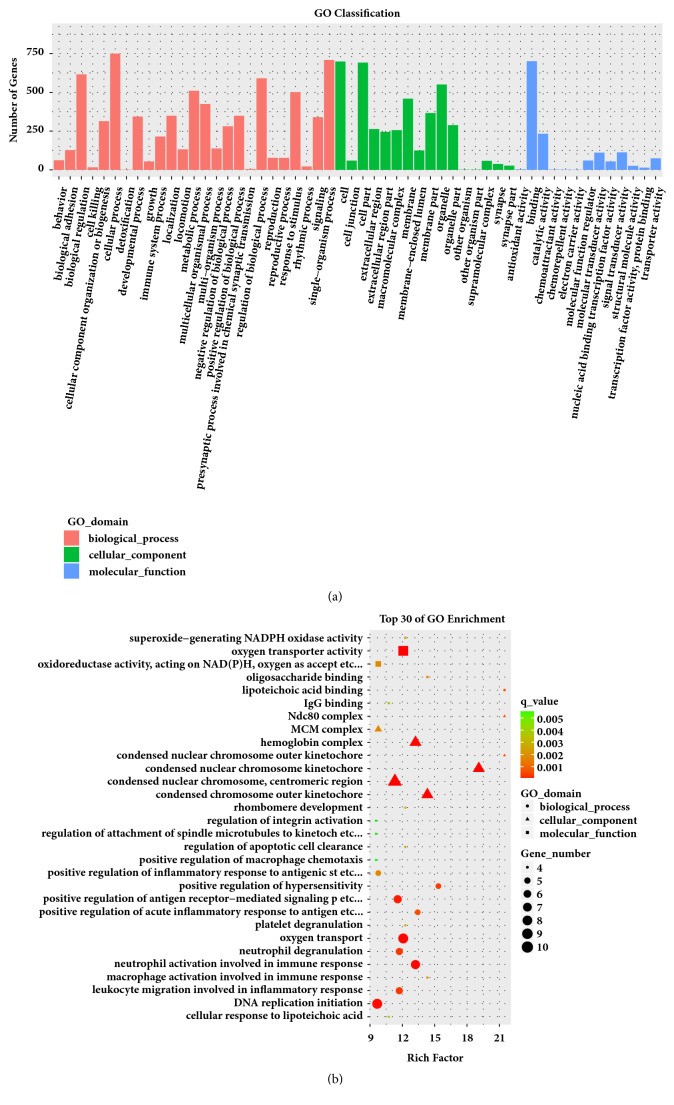
Gene Ontology (GO) enrichment analysis results for all DEGs. (a) Classification of GO enrichment terms. (b) Top 30 classes of GO enrichment terms.

GO enrichment analysis was performed with 1,007 DEGs in clusterProfiler. The DEGs between the MCAO and control groups were enriched to 54 subclasses of GOs, and the top 30 subclasses are shown in Figure 2(a). The top 10 enriched GO biological processes were immune system process, immune response, cell activation, leukocyte migration, response to external stimulus, cell migration, response to wounding, inflammatory response, cell adhesion, and regulation of immune system process. The top 10 enriched cellular components were extracellular space, chromosome, cell surface, extracellular region, nucleosome, vesicle, external side of plasma membrane, kinetochore, condensed chromosome kinetochore, and protein-DNA complex. The enriched GO molecular functions were protein binding (GO:0005515), protein complex binding (GO:0032403), protein heterodimerization activity (GO:0046982), oxygen transporter activity (GO:0005344), lipid binding (GO:0008289), protein dimerization activity (GO:0046983), oxygen binding (GO:0019825), glycosaminoglycan binding (GO:0005539), cytokine receptor activity (GO:0004896), and iron ion binding (GO:0005506). These data are shown in Figure 2.

### 3.3. Pathway Enrichment Analysis of DEGs

**Figure 3:**
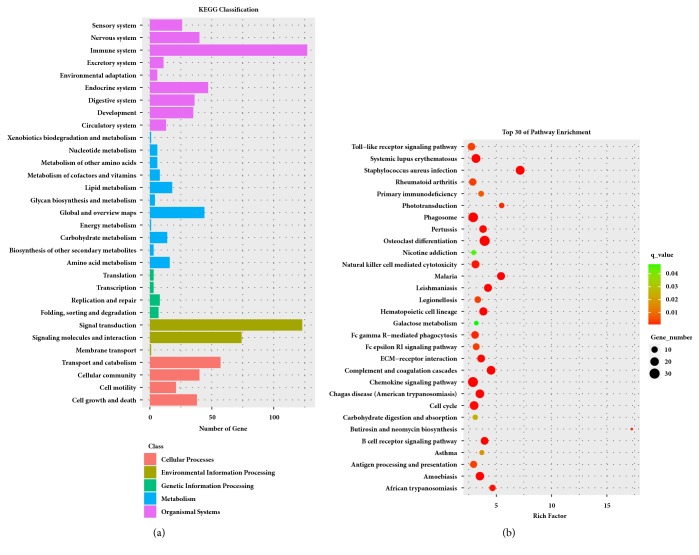
KEGG pathway enrichment analysis results for all DEGs. (a) Classification of pathway enrichment terms. (b) Top 30 classes of pathway enrichment terms.

Pathway enrichment analysis of DEGs could provide further insight into the function of genes and their interactions. The DEGs between the MCAO and control groups were enriched to 31 subclasses of pathways in 5 broad categories (cellular processes, genetic information processing, organic systems, metabolism, and environmental information processing) after these were analyzed using the KEGG database (Figure 3(a)). We performed KEGG pathway enrichment analysis for DEGs and found 237 pathway terms including 77 that were significant (p< 0.05). The top 10 pathways with the greatest enrichment were* Staphylococcus aureus* infection (rno05150), osteoclast differentiation (rno04380), complement and coagulation cascades (rno04610), malaria (rno05144), leishmaniasis (rno05140), chemokine signaling pathway (rno04062), Chagas disease (American trypanosomiasis) (rno05142), hematopoietic cell lineage (rno04640), amoebiasis (rno05146), and phagosome (rno04145). The top 30 enrichment pathways are presented in Figure 3(b).

### 3.4. PPI Network

**Figure 4:**
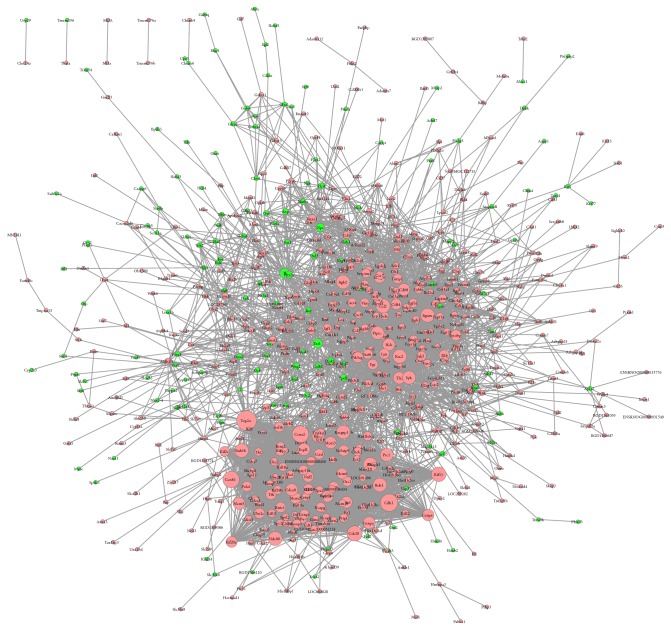
PPI network of the DEGs. Node size is related to node degree. Pink and green nodes denote up- and downregulated genes, respectively. PPI: protein-protein interaction; DEGs: differentially expressed genes.

Significantly altered DEGs were used to construct a PPI network based on the STRING database. The network comprises 744 nodes and 5,266 interactions (Figure 4). More than 100 genes of connectivity degrees were >100, and the details of the top 10 genes with connectivity degrees were listed (Table 3), including Top2a (topoisomerase [DNA] II alpha, degree=121, FC=38.80), Cdk1 (cyclin-dependent kinase 1, degree=104, FC=25.09), Ccna2 (cyclin A2, degree=98, FC= 28.73), Ccnb1 (G2/mitotic-specific cyclin-B1, degree=96, FC=8.072), Th (tyrosine hydroxylase, degree=46, FC=0.03147), and Esr1 (estrogen receptor 1, degree=18, FC=0.2192).

**Figure 5:**
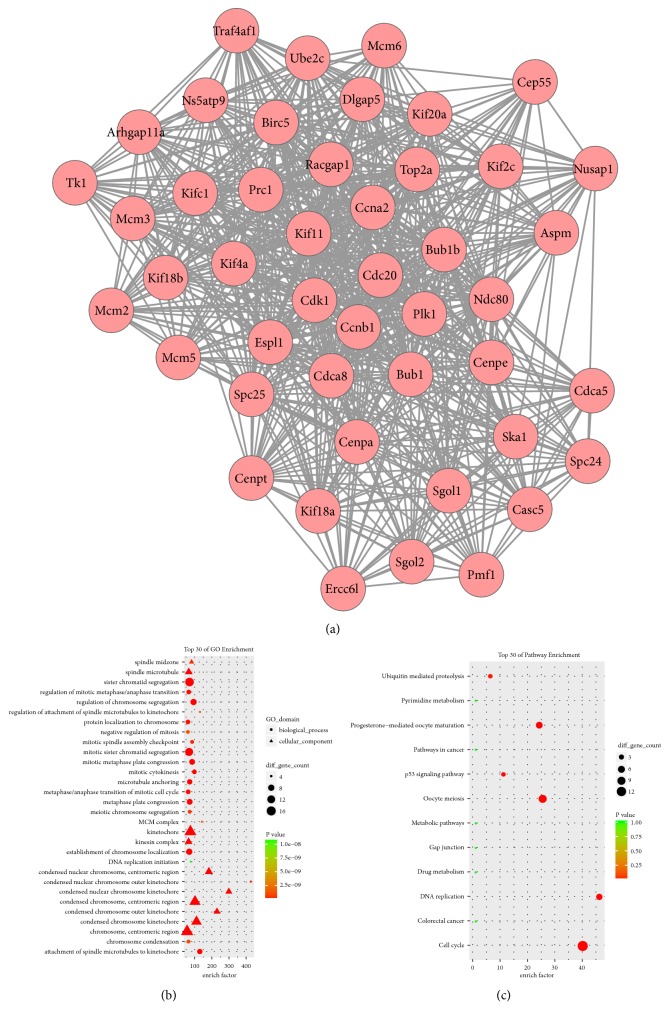
Network, GO enrichment, and pathway enrichment of DEGs involved in module 1. (a) Significant module 1 of the PPI network. (b) Classification of GO enrichment terms. (b) KEGG pathway enrichment terms. Pink and green nodes denote up- and downregulated genes, respectively. PPI: protein-protein interaction; DEGs: differentially expressed genes.

**Figure 6:**
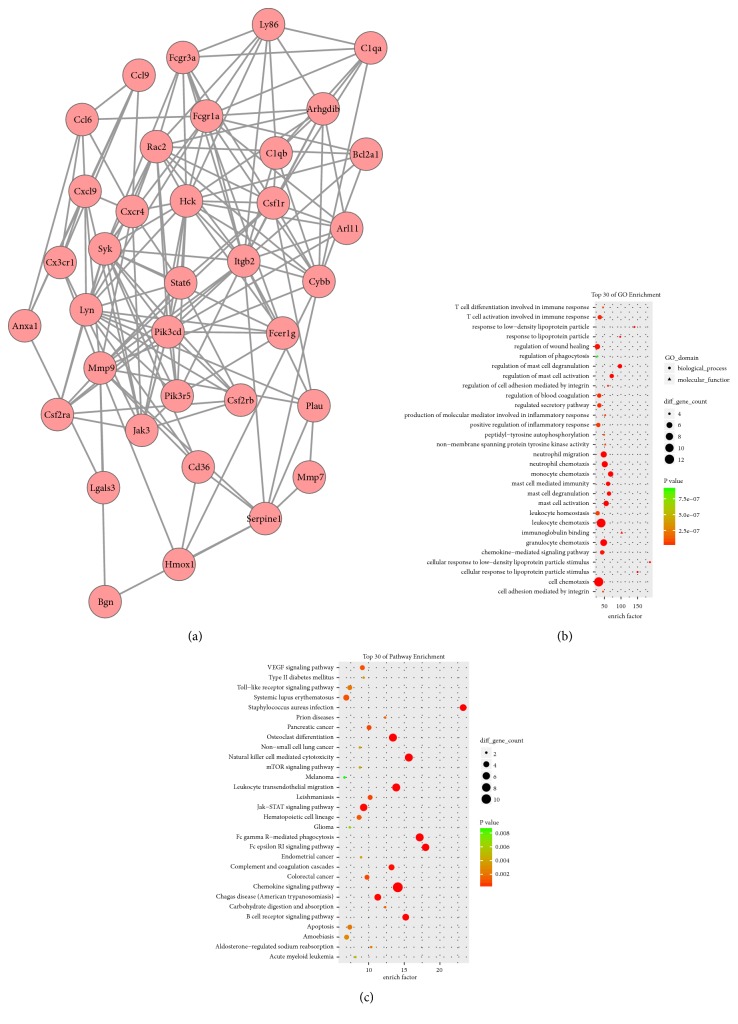
Network, GO enrichment, and pathway enrichment of DEGs were involved in module 2. (a) Significant module 2 of the PPI network. (b) Classification of GO enrichment terms. (b) KEGG pathway enrichment terms. Pink and green nodes denote up- and downregulated genes, respectively. PPI: protein-protein interaction; DEGs: differentially expressed genes.

**Figure 7:**
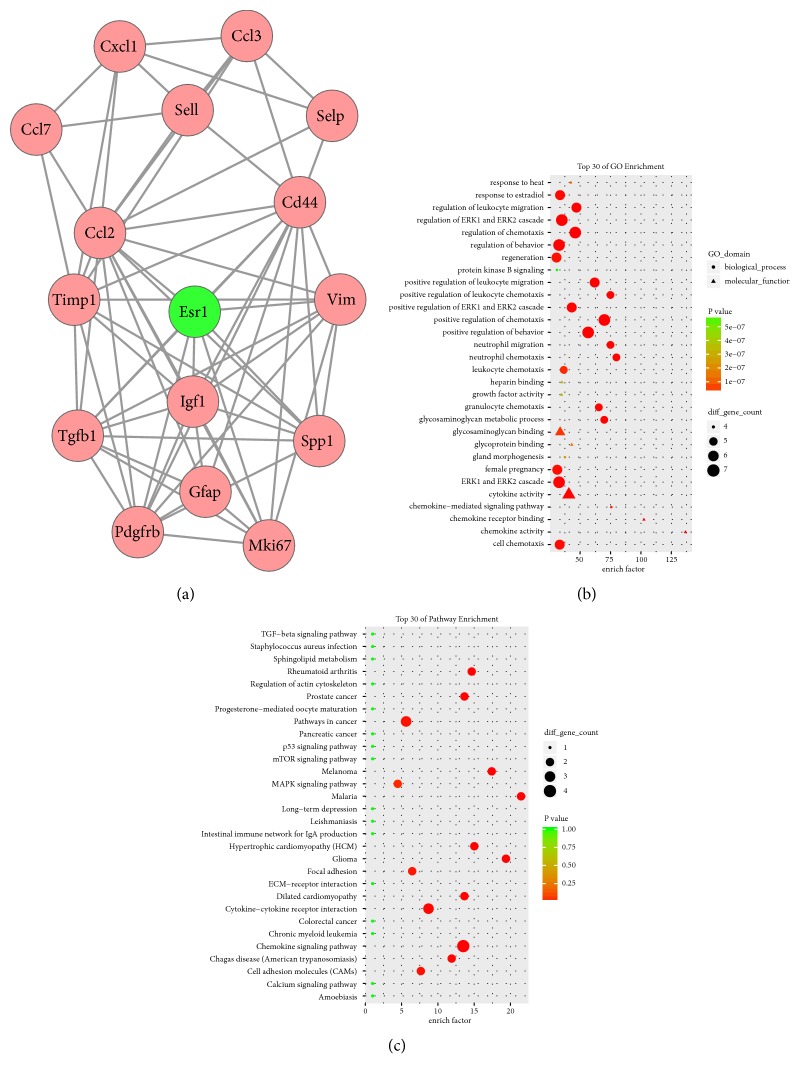
Network, GO enrichment, and pathway enrichment of DEGs were involved in module 3. (a) Significant module 3 of the PPI network. (b) Classification of GO enrichment terms. (b) KEGG pathway enrichment terms. Pink and green nodes denote up- and downregulated genes, respectively. PPI: protein-protein interaction; DEGs: differentially expressed genes.

**Figure 8:**
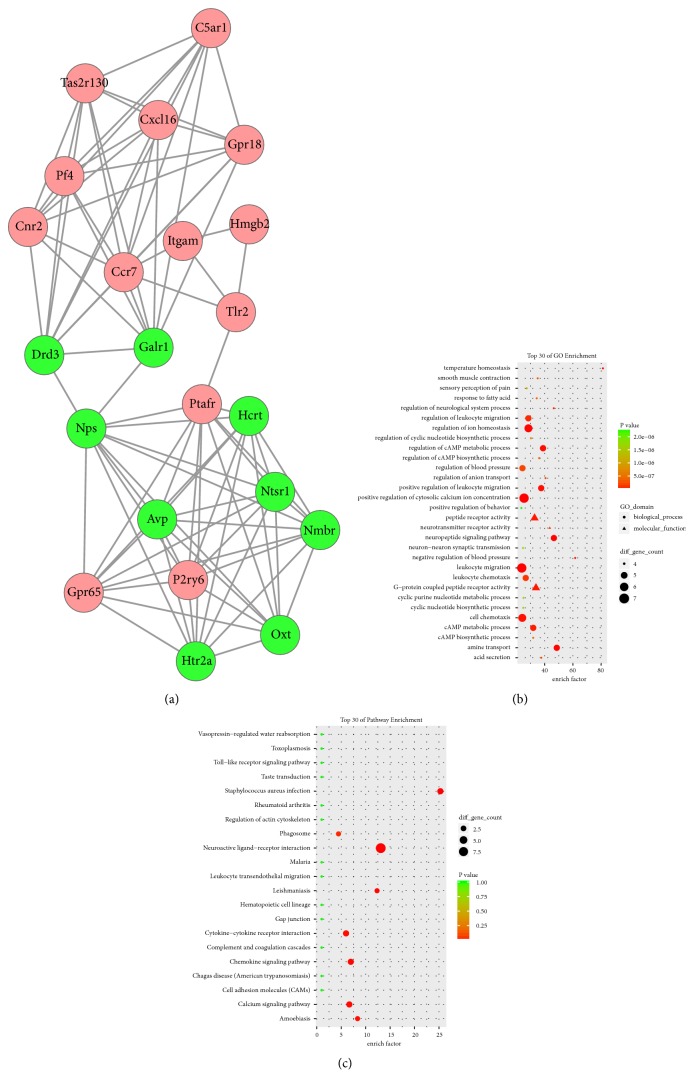
Network, GO enrichment, and pathway enrichment of DEGs involved in module 4. (a) Significant module 4 of the PPI network. (b) Classification of GO enrichment terms. (b) KEGG pathway enrichment terms. Pink and green nodes denote up- and downregulated genes, respectively. PPI: protein-protein interaction; DEGs: differentially expressed genes.

A total of 25 modules were obtained using default criteria in MCODE. Modules were listed in descending order by MCODE score. Four modules with MCODE score ≥4 and nodes ≥10 were named modules 1, 2, 3, and 4. These were selected for module network visualization, GO enrichment analysis, and KEGG pathway enrichment analysis (Figures 5–8). Most DEGs in these modules were upregulated in the MCAO group, especially in modules 1 and 2.

### 3.5. qRT-PCR Verification of the DEGs

**Figure 9:**
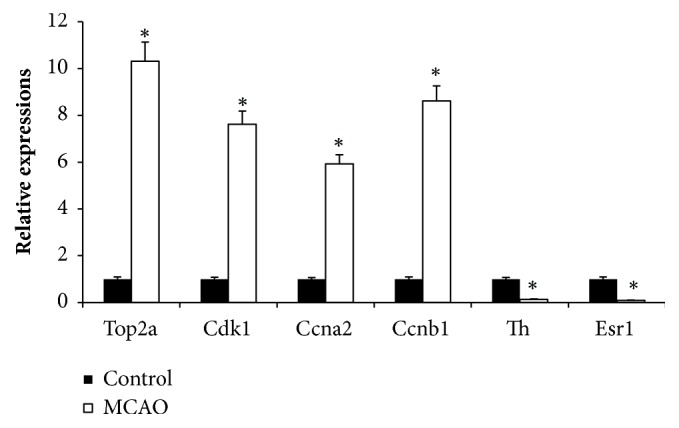
qRT-PCR verification of DEGs. Brain tissue expression of six genes was detected by qRT-PCR and is shown as expression fold changes. GAPDH was the internal control.

Top2a, Cdk1, Ccna2, and Ccnb1 were overexpressed in the MCAO group (Figure 9). Th and Esr1 had lower expression in the MCAO for both RNA-seq and qRT-PCR. We successfully verified that the RNA-seq and qRT-PCR results were consistent, indicating that the RNA-seq results were reliable. The primers are listed in Table 4.

## 4. Discussion

We analyzed the protein-coding mRNA expression profiles in cerebral hemispheres from experimental and control rats by high-throughput RNA-seq. This identified 1,007 DEGs, which were subjected to GO, KEGG pathway, PPI, and PPI module analysis to provide a better understanding of pathological mechanisms that occur after MCAO.

### 4.1. Cell Cycle

Pathway enrichment analysis identified 21 DEGs enriched for the cell cycle (P value=1.25E-05), namely, Mcm6, Ttk, Bub1b, Cdkn2c, Ccnb1, Bub1, Plk1, Orc1, Tgfb1, Mcm3, Cdc6, Mcm5, Pttg1, Cdc20, Chek2, Ccnd1, Mcm2, Espl1, Rbl1, Cdk1, and Ccna2. Most were upregulated in MCAO. Following Cyclin A (Ccna2, FC= 28.73) or B (Ccnb1, FC=8.07) binding and activating cyclin-dependent kinase 1 (Cdk1, FC=25.09), Cdk1 phosphorylates key substrates leading the cell to G2-phase arrest, M-phase, and cytokinesis promotion. Some studies reported that Cdk1 activation is involved in multiple neuronal death by activating phosphorylation of Bad27 (a proapoptotic protein) or inhibitory phosphorylation of Bcl-xL, Bcl-2, and Mcl-1, which are antiapoptotic [28, 29]. Others described similar results in transient ischemia [28, 30]. Besides the Bcl-2 family, Cdk1 also phosphorylates the transcription factor FOXO1, which is also implicated in cell death [31]. We hypothesize that during MCAO, Cdk1 induces ischemic neuronal death by Bcl2 family or Foxo1 promoting signaling pathways.

### 4.2. Inflammatory Response

GO enrichment analysis revealed that more than 78 DEGs are involved in the inflammatory response (p=0.0078), including 75 that were upregulated. Several inflammatory response pathways were activated like complement and coagulation cascades, chemokine signaling pathway, natural killer cell-mediated cytotoxicity, and leukocyte transendothelial migration. Dozens of cytokines were dysregulated. The macrophage recruitment factor CCL2 [32–34] was upregulated, and CD68 and CD163 were dysregulated. Therefore, we hypothesize that MCAO could lead to the recruitment and induction of macrophages, natural killer cells, and leukocytes. The specific cell subsets will require further verification.

### 4.3. Neuroactive Ligand-Receptor Interaction

Pathway enrichment analysis showed that 27 DEGs were involved in the neuroactive ligand-receptor interaction (p=0.00832); 10 and 17 were up- and downregulated, respectively. Upregulated receptors in MCAO were Htr2b, Gabrr1, C5ar1, Cnr2, P2ry6, Htr2a, Galr1, C3ar1, Glra1, and Mc3r. Downregulated receptors in MCAO were Gabrr3, Gabrq, Brs3, Gabra6, Cckar, Trhr, Ptafr, Glra2, Chrnb4, Gpr50, Gabre, Tspo, P2rx2, Ntsr1, Chrna6, Calcr, and Drd3. Lan et al. found decreased serotonin receptor expression in MCAO rats, which was improved with treadmill exercise [35]. We found that Htr2a and Htr2b were upregulated in MCAO group. We hypothesize that MCAO could induce Htr2a and Htr2b overexpression leading to excitability in the early stage of ischemia. Other studies have shown suppression of GABAA and glycine receptors in rats with MCAO, and receptor agonists could improve it [36, 37]. Our results show that some subunits of GABBA (e.g., Gabrr3, Gabrq, Gabra6, Gabre, and Gabrr1) were dysregulated following MCAO.

## 5. Conclusions

By high-throughput RNA-seq, we analyzed the protein-coding mRNA expression profile in control and MCAO groups in brain tissue. And some GOs, pathways, and genes that were identified could play key roles with MCAO. These findings may help us to understand the underlying mechanism of protein-coding mRNAs with MCAO.

## Figures and Tables

**Table 1:** Detailed information of the top 20 upregulated mRNAs in MCAO brain.

gene id	gene name	log2FC	P value
ENSRNOG00000051911	Rbp3	9.837019121	2.34E-05
ENSRNOG00000011672	Tph1	8.512322575	8.73E-05
ENSRNOG00000009907	Mmp8	8.080531694	8.84E-05
ENSRNOG00000010478	LOC299282	7.867067702	3.61E-05
ENSRNOG00000015336	Isl2	7.734722642	0.001563164
ENSRNOG00000007889	Aipl1	7.722506427	0.000503613
ENSRNOG00000000507	Tulp1	7.615402628	0.002259611
ENSRNOG00000007178	Cd8a	7.570686941	0.000201036
ENSRNOG00000007159	Ccl2	7.440623365	5.30E-05
ENSRNOG00000061895	LOC690045	7.288812262	0.000261728
ENSRNOG00000019296	Gnat2	7.142710312	0.000377842
ENSRNOG00000045729	AC117058.1	7.138284224	0.000129134
ENSRNOG00000014787	Cabp5	7.034279622	0.003090557
ENSRNOG00000022859	Trem1	6.919106867	0.003415102
ENSRNOG00000009334	Knstrn	6.883192814	3.60E-05
ENSRNOG00000006365	Asb15	6.822955248	0.004125641
ENSRNOG00000017625	Htr2b	6.556162157	0.003028595
ENSRNOG00000059237	AABR07068851.1	6.516677359	0.005448754
ENSRNOG00000046216	RGD1561778	6.269597389	0.001656204
ENSRNOG00000046962	Pde6g	6.208987117	0.000981813

**Table 2:** Detailed information of the top 20 downregulated mRNAs in MCAO brain.

gene id	gene name	log2FC	P value
ENSRNOG00000008890	Slc18a2	-4.443186456	0.00106392
ENSRNOG00000059292	AABR07064373.1	-4.454664062	0.012966988
ENSRNOG00000012647	Nkx2-4	-4.580004547	0.013461308
ENSRNOG00000005367	Slc12a1	-4.940598177	0.001152078
ENSRNOG00000020410	Th	-4.990029512	0.000109126
ENSRNOG00000024729	Pax5	-4.996430332	0.027951932
ENSRNOG00000003880	Tph2	-5.191719646	0.00042696
ENSRNOG00000060020	C1ql4	-5.489455912	0.023686047
ENSRNOG00000003695	Mgat4d	-5.785311542	0.000949915
ENSRNOG00000011335	Gpr50	-5.787166984	0.005797422
ENSRNOG00000004451	Mc3r	-5.997698916	0.001320591
ENSRNOG00000016613	Hoxc4	-6.046260393	0.00436611
ENSRNOG00000010053	Calcr	-6.355736461	6.56E-07
ENSRNOG00000003476	Slc6a4	-6.847488383	0.000207349
ENSRNOG00000007608	Fezf1	-7.085814019	4.92E-05
ENSRNOG00000037600	Sim1	-9.291469805	1.05E-08
ENSRNOG00000017302	Slc6a3	-9.332130194	2.70E-05
ENSRNOG00000021225	Oxt	-9.69339756	7.12E-11
ENSRNOG00000021229	Avp	-9.81990178	1.10E-08
ENSRNOG00000004632	Pmch	-10.02651105	4.67E-05

**Table 3:** Top 10 degrees of up- and downregulated DEGs.

Gene_name	log2FC	P value	Up/down	PPI_node_degree
Top2a	5.278098	2.91E-05	UP	121
Cdk1	4.649292	0.000415	UP	104
Ccna2	4.844517	4.87E-05	UP	98
Ccnb1	3.01E+00	0.005115	UP	96
Cdc20	2.615553	0.007635	UP	86
Kif11	4.063773	6.35E-05	UP	83
Ccl2	7.440623	5.3E-05	UP	79
Itgam	2.150151	0.004347	UP	78
Ndc80	3.562942	0.005167	UP	78
Plk1	2.267746	0.013009	UP	78
Th	-4.99003	0.000109	DOWN	46
Esr1	-2.18964	0.005317	DOWN	44
Nps	-∞	0.035781	DOWN	36
Htr2a	-1.0935	0.002992	DOWN	26
Ppef1	-2.41715	0.038577	DOWN	24
Prkg2	-1.00732	0.029108	DOWN	24
Pcsk1	-1.04925	0.04309	DOWN	22
Calb2	-2.19721	0.005492	DOWN	20
Ret	-1.7584	0.004092	DOWN	20
Avp	-9.8199	1.1E-08	DOWN	19

**Table 4:** Primer sequences.

Gene	Sequences	PCR product length (bp)
GAPDH	F: CCTGGTATGACAACGAATTTG	131
	R: CAGTGAGGGTCTCTCTCTTCC	
Top2a	F: CAGCAGAAGGTCCCAGAAGA	100
	R: GGTAGTTGAAGGTCGGTCCA	
Cdk1	F: GTACGGCAATCCGGGAAATC	98
	R: GAGATACAGCCTGGAGTCCT	
Ccna2	F: AGCTCTCTACACAGTCACAGG	106
	R: GGTCTGGTGAAGGTCCATGA	
Ccnb1	F: CAGGGTCACTAGGAACACGA	121
	R: AGCAGTTCTCGATCTCAGCA	
Th	F: GTCGGAAGCTGATTGCAGAG	129
	R: TAGCATAGAGGCCCTTCAGC	
Esr1	F: CCAGCTCCTCCTCATCCTTT	101
	R: GGTCATAGAGAGGCACGACA	

## Data Availability

The data used to support the findings of this study are available from the corresponding author upon request.
